# The Typhoid Epidemic in Corning

**Published:** 1912-06

**Authors:** Frank S. Swain

**Affiliations:** Health Officer, Corning, N. Y.


### The Typhoid Epidemic in Corning

DR. FRANK S. SWAIN, Health Officer, Corning, N. Y.

FROM the 15th of April to the present time, May 15, 1912, the city of Corning has been in the clutches of a typhoid fever epidemic. It has been of no mean proportion, ninety-five cases in a population of 13,700 having been reported to the Health Department during that time. Five deaths have resulted, four of which were among children between the ages of five and fifteen years.

Almost everyone is of the opinion that polluted city water was the cause of the epidemic. This idea is confirmed by the subsequent events, coupled with the results of expert investigation.

The city is furnished with water that comes from springs which are situated in the lowest part of the city. These empty into an impounding basin from which the water is pumped through the mains, all surplus water going into two reservoirs, one on each side of the city. Both of the reservoirs are enclosed with cement walls and roof. The idea in covering them was to prevent the growth of algae on the water, which is extremely hard in character.

Near the impounding basin is located one of the city parks, in which there is an artificial lake into which the overflow from the impounding basin is emptied. In the overflow pipe there is a shut off valve which is supposed to be closed during high water. The nature of the soil in the vicinity, and covering a large area about the impounding basin, is of a very coarse gravel, possessing little, if any filtering properties.

On or about March 29th the Chemung river, which runs through the city and also near the lower end of the park, became very high on account of the heavy rains. The result was that the water in this little lake overflowed its bank and soaked through the soil surrounding the immediate vicinity of the impounding basin. It has been alleged that the polluted water backed through the overflow pipe into the impounding basin. Be that as it may, on the morning of March 30th, Dr. Lundblad, the county bacteriologist, who examines the water daily, discovered its contamination and at once notified the health officer who in turn requested the superintendent of the water works to immediately treat the water with hypochlorite of lime, as has been the custom on several occasions during the past three years (about one-twentieth of a grain per gallon being used). The next day sickness prevailed throughout the city. Some fifteen hundred people were affected, nausea, vomiting, severe abdominal pains and diarrhoea being the characteristic symptoms and lasting from one to three days. It seemed to have played havoc among the people, the symptoms being more pronounced and the suffering more intense among the little ones. After about two weeks typhoid developed generally throughout the city, no part escaping and all classes being affected. Many of those who experienced bowel trouble immediately after the water became polluted afterwards developed typhoid, but there were a few who had no diarrhoeal condition at all, constipation taking its place.

After some two weeks, Doctor Lundblad of the laboratory advised that the water from the public tap was free of any contamination, also the water in the impounding basin. However, the public was not advised for at least a week afterwards that the water has been declared safe for domestic purposes.

During the latter part of April the reports of the typhoid cases became so numerous and continued to such a marked degree, as high as eight in one day, that the health officer deemed it advisable to call upon the State Health Department for assistance.

Dr. Eugene H. Porter, Commissioner of Health of the State of New York, sent Mr. Theodore Horton, State Sanitary Engineer here to investigate the cause of the epidemic and to make such suggestions as he deemed pertinent to improve the situation. Mr. Horton stated that there was no question but that the source of the water supply became polluted and he directed the board of public works, which has charge of the water department of the city, to empty both reservoirs at once, treat them with hypochlorite of lime and then have them refilled, also that all dead ends in the water mains be opened and thoroughly flushed. On Thursday, May 9th, the water in the reservoir on the north side of the river was found to be contaminated. Immediate steps were taken to have the same purified.

The citizens have been aroused to the seriousness of the situation. Doctors, nurses, the families of those afflicted, milkmen, school children, and, in fact, everyone has been advised to exercise every precaution possible to stamp out the disease and to prevent further cases developing. It is being done.

A word as to the way the most of the typhoid cases are running. In the majority of the cases it prevails among children. The temperature will be normal in the morning and in the afternoon it will have gone up to 104, a pronounced headache accompanying, the usual step ladder temperature being absent. In many cases the fever has not been severe and a great number are now convalescing. The situation is well in hand now, but five cases having been reported during the past five days.

The old saying, "It is a bad wind that does not blow some good" will apply to this epidemic. Corning will be furnished with a new supply of water, one what will be pure and wholesome at all times.

